# A Rare Case of Primary Mesenteric Liposarcoma

**DOI:** 10.7759/cureus.38329

**Published:** 2023-04-30

**Authors:** Priya Ahire, Aldrin L Myrthong, Suresh Mahankudo, Mukund B Tayade, Sumit Boricha

**Affiliations:** 1 General Surgery, Grant Government Medical College and Sir JJ Group of Hospitals, Mumbai, IND; 2 General Surgery, Grant Government Medical College and JJ Group of Hospitals, Mumbai, IND

**Keywords:** excision, chemotherapy, dedifferentiated, mesenteric liposarcoma, primary

## Abstract

Primary mesenteric liposarcoma is a rare soft tissue malignant neoplasm. The authors present a case of a 42-year-old male with pain in the abdomen and abdominal mass which showed a desmoid tumor on biopsy and CT shows a mesenteric mass present. The patient underwent exploratory laparotomy and a large tumor was excised. The specimen was sent for histopathology and showed dedifferentiated liposarcoma of the mesentery. Immunohistochemistry showed the tumor cells are diffusely positive for mouse double minute 2 (MDM2), p16, and show patchy positivity for the cluster of differentiation (CD) 34. The cells are negative for smooth muscle actin (SMA), desmin, S100, and ckit. After the surgery, the patient recovered well and was given adjuvant chemotherapy with doxorubicin, ifosfamide, and mesna. The patient has no signs or symptoms of recurrence to date. In this case, the combination of surgery and chemotherapy has shown to have a good clinical outcome.

## Introduction

Liposarcoma (LPS) is a malignant soft-tissue tumor whose incidence peaks in the fourth to sixth decade of life [1] and is usually found in the retroperitoneum, extremities, or cervical area [2]. Although liposarcomas are common tumors of the retroperitoneum, primary mesenteric liposarcomas are extremely rare neoplasms. It was not until 1951 that it was first described in the literature by Manson [3] and the presence of only 23 well-described reported cases of mesenteric liposarcomas in literature is a testimony of this fact [4]. They are known to grow relentlessly into large tumors and become symptomatic due to compression on adjacent organs. There is a lack of standard guidelines on its management due to its rare occurrence. WHO classifies liposarcoma into various subtypes: well-differentiated, pleomorphic, myxoid, round cell, and dedifferentiated [5]. This article reports a case of dedifferentiated primary mesenteric liposarcoma where the patient was evaluated clinically and radiological investigations were done pre-operatively followed by adjuvant chemotherapy after the surgery.

## Case presentation

A 42-year-old male patient presented with a history of pain in the left side of the abdomen for the past two months. There was no history suggestive of abdominal mass, significant weight loss, anorexia, gastric outlet obstruction, altered bowel habits, malena, or urinary complaints. The patient had a history of pulmonary tuberculosis five years back for which he received treatment. The patient also underwent right inguinal hernioplasty four years back. He is a chronic tobacco chewer for the past 15 years.

**Figure 1:**
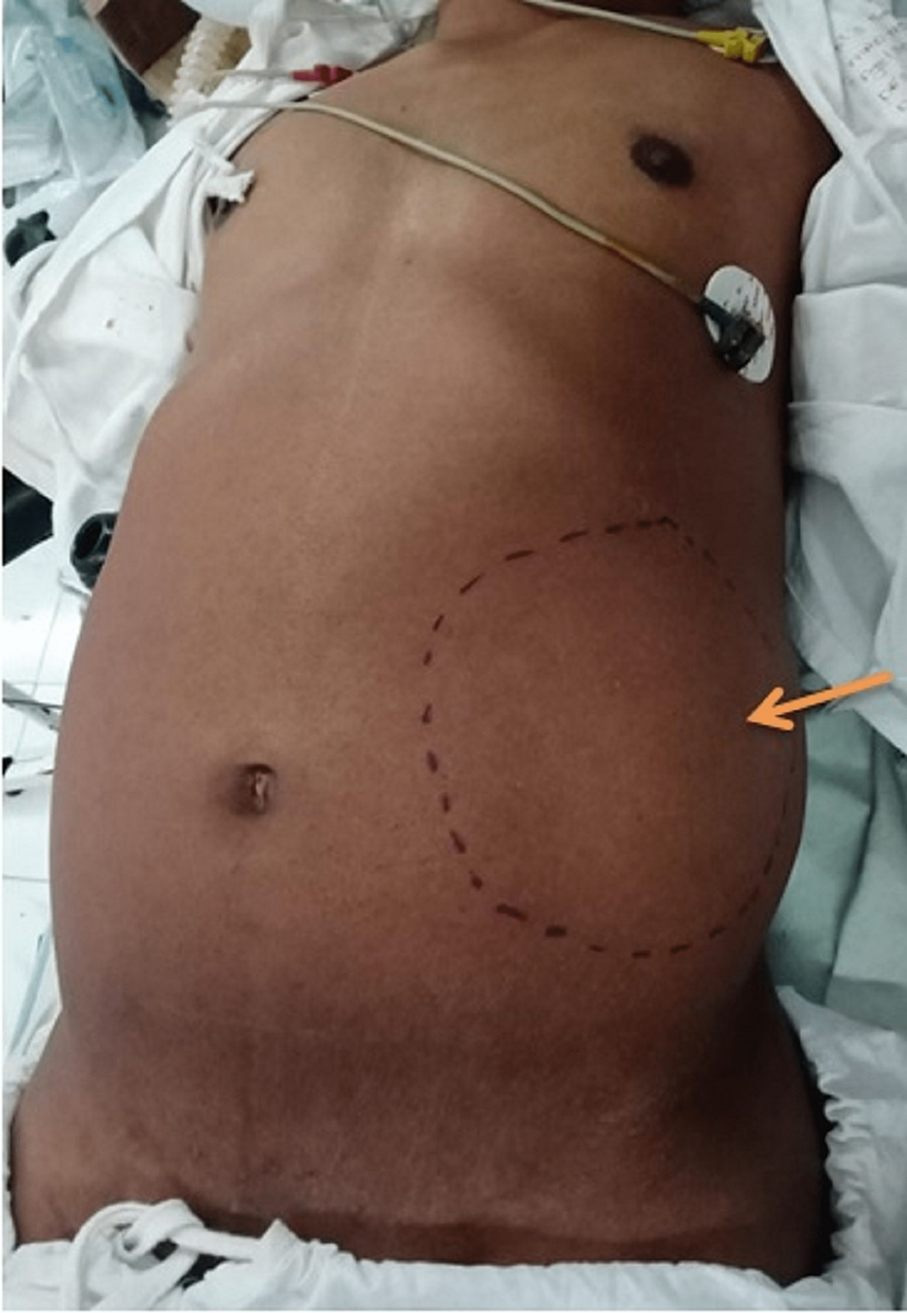
Mass in the left hypochondriac and left lumbar region with margins demarcated Orange arrow: mass with palpable margins

On examination of the abdomen, a large, intra-abdominal, mobile mass was found which is well defined, hard in consistency, non-tender, approximately 10cm × 9cm in size occupying the left hypochondrium and left lumbar region of the abdomen (Figure 1). There was no sign of ascites.

Ultrasonography of the abdomen revealed a well-defined intra-abdominal solid lesion in the left lumbar region below the inferior pole of the left kidney

**Figure 2:**
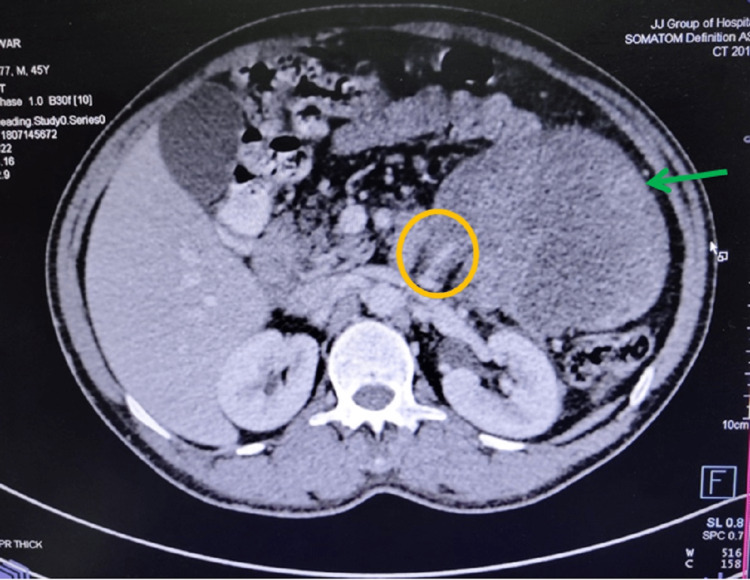
- Axial section of CT abdomen showing the tumour with feeding vessel and its relation with the surrounding structures Green arrow - Heterogeneously enhancing, solid lesion Yellow circle - Feeding vessels

**Figure 3:**
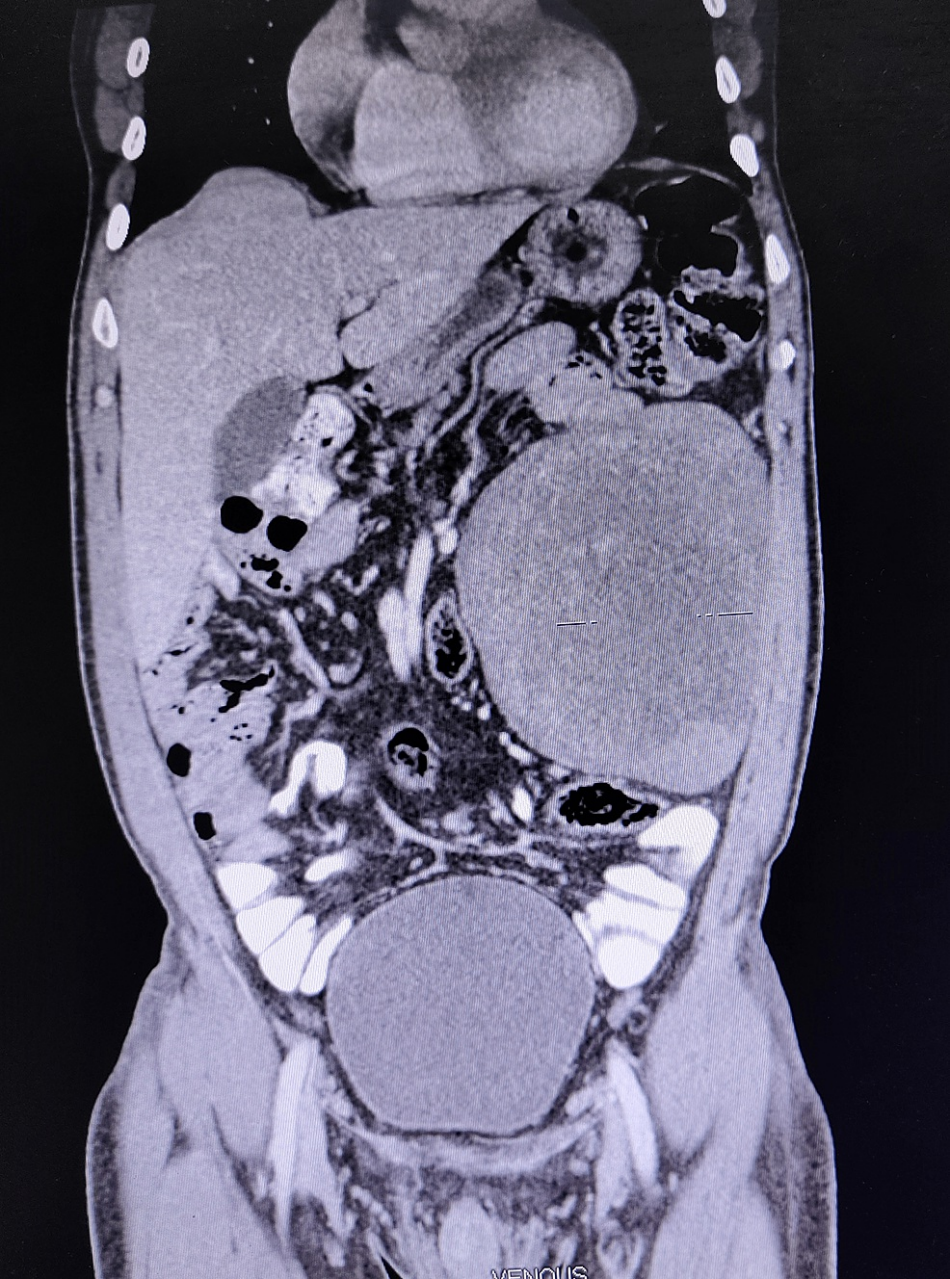
Coronal section of CT abdomen showing the tumour and its relation with the surrounding structures

**Figure 4:**
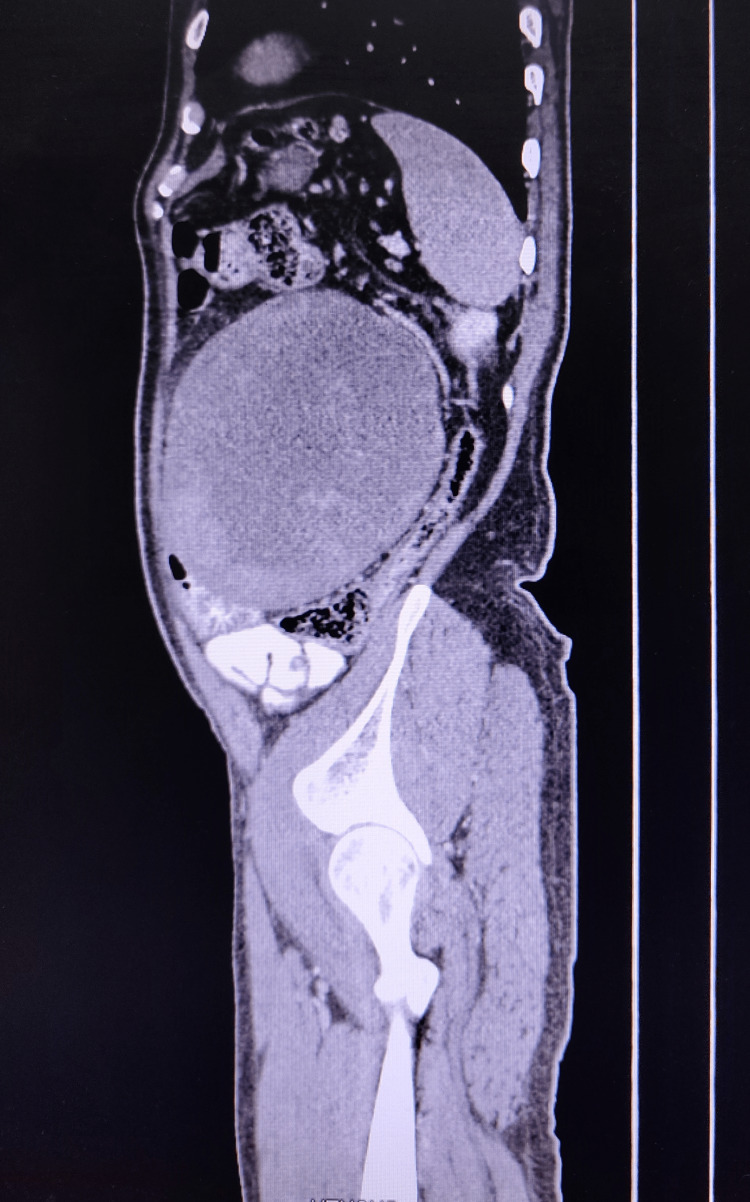
Sagittal section of CT abdomen showing the tumour and its relation with the surrounding structures

A contrast-enhanced CT scan of the abdomen and pelvis was done which showed a well-defined, lobulated, heterogeneously enhancing, solid lesion (HU- 93) measuring 12.6cm × 13cm × 14.7cm noted within the mesentery of the left lumbar region. There is no evidence of early arterial enhancement seen. The lesion was limited anteriorly by the anterior abdominal wall, medially displacing the small bowel loops and posteriorly pushing the descending colon. No calcification was seen (Figures 2-4).

A USG-guided biopsy was taken which showed features suggestive of a myofibroblastic tumor-desmoid tumor.

**Figure 5:**
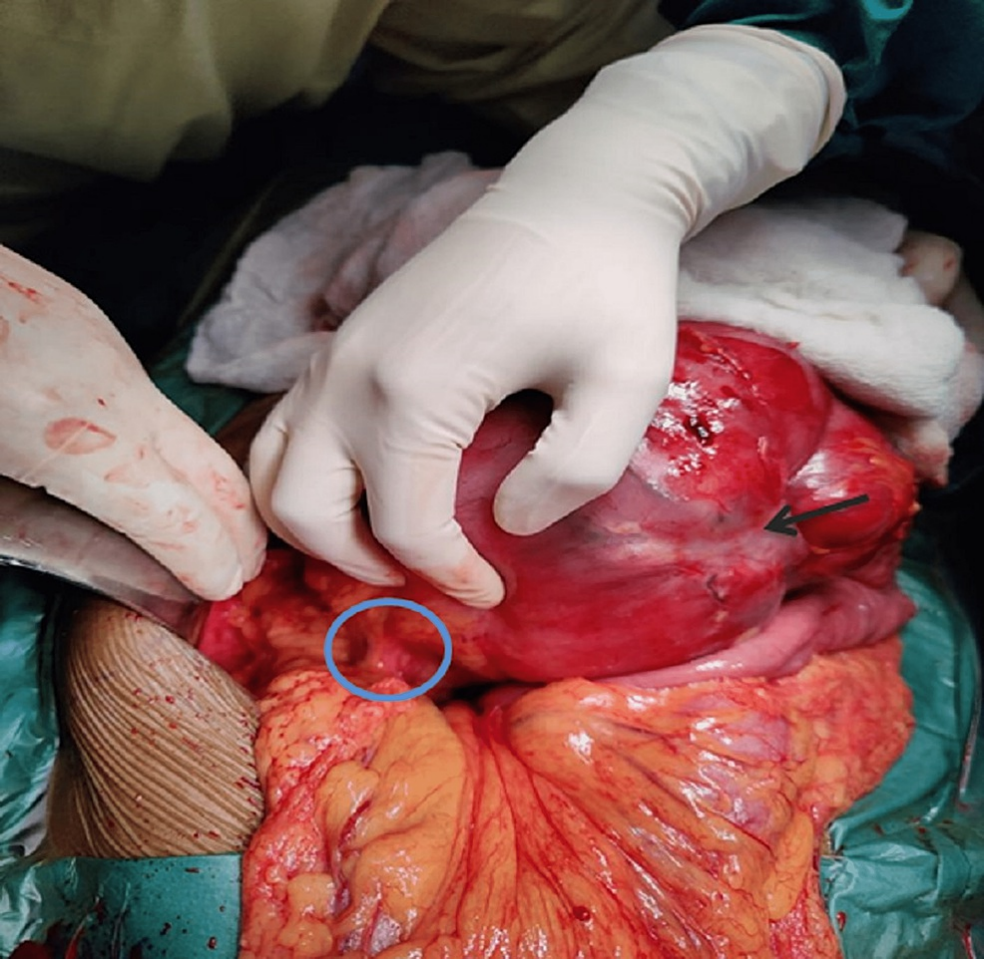
Intraoperative image of the tumor with feeding vessels Blue circle - Feeding vessels, Black Arrow - Tumour

**Figure 6:**
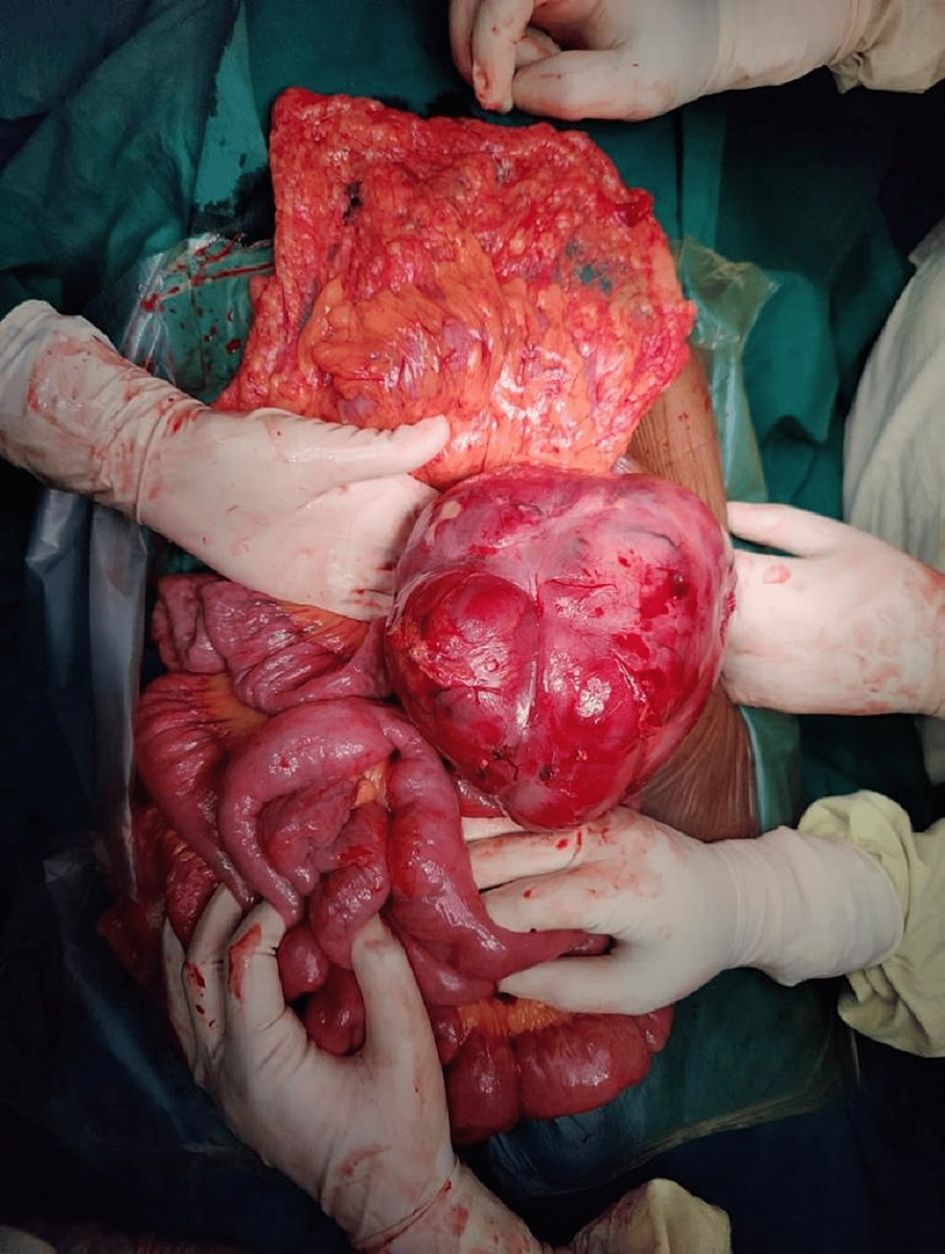
Intra-operative image of the tumour

The patient was taken up for exploratory laparotomy with due fitness for general anesthesia. A large, multilobulated tumor approximately 20cm × 15cm in size was seen arising from the mesentery of the jejunum on the left side of the duodenal-jejunal flexure and aorta with no adhesions to surrounding structures. Feeding vessels of the tumor were identified. The tumor was not invading any vascular structure (Figures 5, 6).

**Figure 7:**
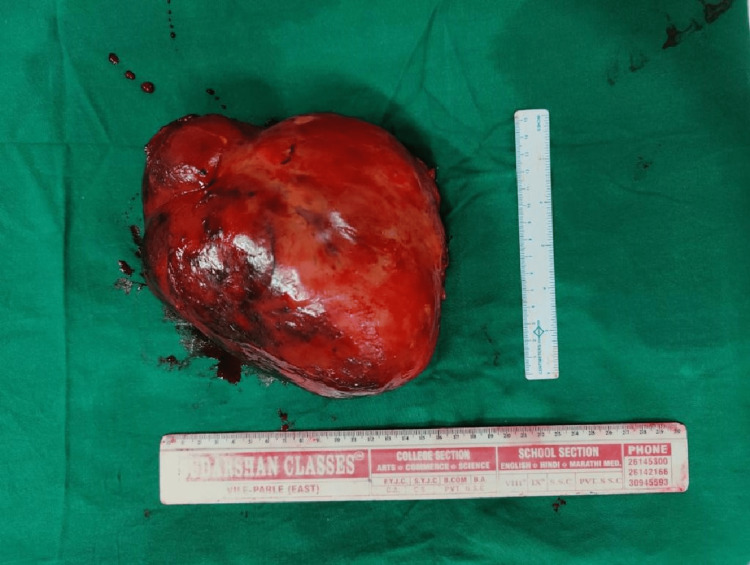
The tumour after excision from the bowel mesentery

Meticulous dissection was done using a harmonic scalpel and monopolar cautery to release adhesions from the tumor. Feeding vessels were identified and ligated and cut. Tumour was excised. The rest of the visualized viscera and bowel were inspected and found to be normal. There was no lymphadenopathy of the superior mesenteric group of lymph nodes or the pre-aortic lymph nodes (Figure 7).

The tumor’s cut surface had a variegated appearance with yellow gritty areas, gelatinous/ mucinous greenish areas, and regions of necrosis. On histopathological examination, the specimen showed features suggestive of dedifferentiated liposarcoma. The sections studied showed a neoplasm composed of spindle cells arranged in interlacing bundles and fascicles. The cells showed moderate nuclear pleomorphism with a vesicular nucleus. Scattered bizarre cells are seen. mitosis (10-12/10hpf) and necrosis noted. The stroma showed myxoid change and delicate branching vessels. The tumor is seen infiltrating the adipose tissue in the periphery. The capsule is seen free of tumor. Focal lymphoid aggregates are noted. On immunohistochemistry, the tumor cells were diffusely positive for mouse double minute 2 (MDM2), p16 and showed patchy positivity for cluster of differentiation (CD) 34 and negative for smooth muscle actin (SMA), Desmin, S100, and ckit.

This patient had an uneventful post-operative period and was discharged on postoperative day seven. The patient has completed five cycles of chemotherapy post-operatively. The patient was given an injection of doxorubicin 40mg, an injection of ifosfamide 4gm, and mesna 960mg on days one, two, and three of each cycle of every 21 days. The patient is currently in the sixth month of follow-up with no symptoms or signs of recurrence.

## Discussion

Primary mesenteric liposarcomas are rare neoplasms that are similar to liposarcomas occurring in other sites of the body: these lesions mostly occur in the fifth to seventh decade of life with males getting slightly more affected [6-8].

The clinical presentation depends on the size and location of the tumor. They become clinically symptomatic in view of sheer size or compression on adjacent organs. The finding of a freely mobile mass on physical examination is frequent and can help in the diagnosis [6,7]. Patients may present with complaints of gradual distension of the abdomen, pain in the abdomen, loss of weight, early satiety, and abdominal masses which are freely mobile. These tumors can rarely cause intussusception, obstruction, acute appendicitis, perforation, or symptoms mimicking diverticulitis [9].

For diagnosis, CT scans can provide important information regarding the size and involvement of adjacent structures as well as tissue characteristics of the tumor [10-13]. The characteristics of liposarcoma on CT images are i) infiltration or poor margination, ii) inhomogeneity, iii) CT Hounsfield units (HU) greater than the patient's normal fats, and iv) contrast enhancement [12]. The degree of histological grade shows a difference in enhancement on CT accordingly [13]. The CT scan of our patient shows a heterogeneously enhancing solid lesion with CT Hounsfield unit greater than the patient's normal fat but the lesion was well-defined and had no contrast enhancement. The degree of histological grade by the difference in enhancement on CT was not appreciable.

In our patient, a CT scan showed a well-defined, lobulated, heterogeneously enhancing, solid lesion likely arising from the mesentery. There is no evidence of early arterial enhancement seen. The lesion was limited anteriorly by the anterior abdominal wall, medially it was displacing the small bowel loops and posteriorly it was pushing the descending colon. No calcification was seen.

WHO classifies liposarcoma into various subtypes: well-differentiated, pleomorphic, myxoid, round cell, and dedifferentiated [5]. The most common type is myxoid liposarcoma, occurring in approximately 50% of cases, this is followed by well-differentiated liposarcoma found in 25% of the cases and has the best prognosis. Dedifferentiated liposarcoma is more aggressive than pure well-differentiated liposarcoma and has a local recurrence rate of 41%, a metastasis rate of 17%, and disease-related mortality of 28% [14]. In our patient, a histopathological examination showed dedifferentiated liposarcoma, and hence chemotherapy was planned for him to prevent recurrence owing to the more aggressive type of liposarcoma detected. 

The treatment for mesenteric liposarcoma consists of a tumor excision with clear margins followed by adjuvant chemotherapy and/or radiotherapy in patients who are at high risk of recurrences such as large tumor size (>5cm) or poorly differentiated tumors [15]. It is reported that neoadjuvant chemotherapy may play a role in the reduction of the primary tumor size so that the tumor can be resectable without the need for resection of the adjacent organs [15].

The drug used for chemotherapy is doxorubicin. It is shown in 14 randomized trials that adjuvant doxorubicin-based chemotherapy significantly decreases the local and distant recurrence and tends to improve the overall survival rate of patients [16]. Another active chemotherapeutic drug used in the treatment of sarcomas is ifosfamide. A combination of both doxorubicin and ifosfamide has shown a high response rate for primary sarcomas with a high risk for recurrence. Patel et al. showed a response rate of 66% (46-82%, 95% confidence interval) [17] and De Pas et al. reported a response rate of 50% (23-77%, 95% confidence interval) [18]. Many studies have shown that this high-dose combination chemotherapy of doxorubicin and ifosfamide is the most hopeful regimen for soft tissue sarcoma [19]. 

## Conclusions

Primary mesenteric liposarcoma is a rare entitiy. CT and MRI are important radiological investigations in finding the characteristics of the tumor, the tumor size, and the presence of any invasion into the surrounding structures. The tumor is often resectable and the treatment of choice is surgery with resection of the tumor followed by adjuvant chemotherapy and/or radiotherapy. Histological classification and tumor site and size determine the prognosis of the patient while positive surgical margins are associated with an increased risk of local recurrence. The patient should be followed up to assess the role of chemotherapy and to detect recurrence as the rate of recurrence of liposarcomas is high.

## References

[1] Yuri T, Miyaso T, Kitade H, Takasu K, Shikata N, Takada H, Tsubura A (2011). Well-differentiated liposarcoma, an atypical lipomatous tumor, of the mesentery: a case report and review of the literature. Case Rep Oncol.

[2] Dei Tos AP (2014). Liposarcomas: diagnostic pitfalls and new insights. Histopathology.

[3] MA JM (1951). Mesenteric liposarcoma; a rare cause of intestinal obstruction. Br J Surg.

[4] Mokfi R, Boutaggount F, Maskrout M, Rais G (2022). Giant mesenteric myxoid liposarcoma: challenges of diagnosis and treatment. Radiol Case Rep.

[5] DeVita VT, Lawrence TS. Rosenberg SA (2011). Cancer: principles and practice of oncology-advances in oncology. https://books.google.com/books?hl=en&lr=&id=p-kieqvYUtYC&oi=fnd&pg=PT32&dq=Cancer:+Principles+and+Practice+of+Oncology-Advances+in+Oncology&ots=Zm9EssAu16&sig=c4wSTlE9ZAVPGgjygWZZ3lmgTg8#v=onepage&q=Cancer%3A%20Principles%20and%20Practice%20of%20Oncology-Advances%20in%20Oncology&f=false.

[6] Moyana TN (1988). Primary mesenteric liposarcoma. Am J Gastroenterol.

[7] Baldi A, Ganio E, Rosato L (1982). Considerazioni su un caso di liposarcoma primitivo del mesentere (Article in Italian). Arch Sci Med.

[8] Calò PG, Congiu A, Ferreli C, Nicolosi A, Tarquini A (1994). I tumori retroperitoneali primitivi. Nostra esperienza (Article in Italian). Minerva Chir.

[9] Horiguchi H, Matsui M, Yamamoto T, Mochizuki R, Uematsu T, Fujiwara M, Ohse H (2003). A case of liposarcoma with peritonitis due to jejunal perforation. Sarcoma.

[10] Hunter JC, Johnston WH, Genant HK (1979). Computed tomography evaluation of fatty tumors of the somatic soft tissues: clinical utility and radiologic-pathologic correlation. Skeletal Radiol.

[11] Kim T, Murakami T, Oi H (1996). CT and MR imaging of abdominal liposarcoma. AJR Am J Roentgenol.

[12] Friedman AC, Hartman DS, Sherman J, Lautin EM, Goldman M (1981). Computed tomography of abdominal fatty masses. Radiology.

[13] Waligore MP, Stephens DH, Soule EH, McLeod RA (1981). Lipomatous tumors of the abdominal cavity: CT appearance and pathologic correlation. AJR Am J Roentgenol.

[14] Hasegawa T, Seki K, Hasegawa F (2000). Dedifferentiated liposarcoma of retroperitoneum and mesentery: varied growth patterns and histological grades--a clinicopathologic study of 32 cases. Hum Pathol.

[15] Ishiguro S, Yamamoto S, Chuman H, Moriya Y (2006). A case of resected huge ileocolonic mesenteric liposarcoma which responded to pre-operative chemotherapy using doxorubicin, cisplatin and ifosfamide. Jpn J Clin Oncol.

[16] Sarcoma Meta-analysis Collaboration (1997). Adjuvant chemotherapy for localised resectable soft-tissue sarcoma adults: meta-analysis of individual data. Sarcoma Meta-analysis Collaboration, Lancet.

[17] Patel SR, Vadhan-Raj S, Burgess MA, Plager C, Papadopolous N, Jenkins J, Benjamin RS (1998). Results of two consecutive trials of dose-intensive chemotherapy with doxorubicin and ifosfamide in patients with sarcomas. Am J Clin Oncol.

[18] De Pas T, De Braud F, Orlando L (1998). High-dose ifosfamide plus adriamycin in the treatment of adult advanced soft tissue sarcomas: is it feasible?. Ann Oncol.

[19] Frustaci S, Gherlinzoni F, De Paoli A (2001). Adjuvant chemotherapy for adult soft tissue sarcomas of the extremities and girdles: results of the Italian randomized cooperative trial. J Clin Oncol.

